# Informed Contraceptive Decisions: A Qualitative Study of Hispanic Teens in New Mexico

**DOI:** 10.1089/whr.2022.0070

**Published:** 2022-12-13

**Authors:** Mayra Perez, Stephanie Chambers, Venice Ceballos, Allyson Kelley, Jennifer Hettema, Andrew Sussman, Sally Kosnick, Evangelina Moralez-Norris, Samantha Jackson, Melanie Baca

**Affiliations:** ^1^ Community Health Work Initiatives, University of New Mexico, Health Sciences Center, Albuquerque, New Mexico, USA.; ^2^ Department of Family and Community Medicine, University of New Mexico, Albuquerque, New Mexico, USA.; ^3^ Allyson Kelley & Associates PLLC, Albuquerque, New Mexico, USA.; ^4^ LifeStance Health, Inc, Albuquerque, New Mexico, USA.; ^5^ New Mexico GRADS Program, Socorro, New Mexico, USA.

**Keywords:** adolescent, reproductive health, Hispanic, long-active reversible contraception

## Abstract

**Objectives:**

U.S. Hispanic teens experience higher rates of unintended pregnancy than white teens. Limited research has been done to explore the sociocultural factors that impact Hispanic teens and their decisions about birth control and long-acting reversible contraception (LARC). The theory of planned behavior served as a framework for this study and teen perspectives about contraceptive decision making. This study aimed to identify the sociocultural factors that impact Hispanic teens when they make decisions about birth control and LARC.

**Study Design and Methods:**

We interviewed Hispanic teens from school-based health centers in New Mexico during their schedule medical appointments. Interviews were audio recorded, transcribed, and coded using content analysis coding methods and a descriptive qualitative design.

**Results:**

A total of 20 Hispanic teens participated in this study, all were female and between the ages of 14 and 19 years.

**Themes:**

Five themes emerged from the analysis process that impact Hispanic teen contraceptive choice, these are family, religion, culture, peer influence, and other factors.

**Conclusions and Implications:**

Among both LARC and non-LARC groups, peer influence was the most frequently cited reason for contraceptive decision making. Relationships with family were cited as barriers for Hispanic teens, where lack of communication and abstinence-only beliefs made it difficult to seek contraception. Findings demonstrate that teens selected LARCs because of the impacts on menstrual cycles and clinician influence. Teens who did not self-select LARC discussed ease of protection and the utilization of birth control as a transition to LARC.

## Introduction

Nearly half of American teenagers (4 million) are sexually experienced.^1^ In a given year, 6% of American teens become pregnant^2^ and 82% of these pregnancies are unplanned.^3^ Although teen pregnancy has decreased over the past two decades, rates are still much higher than those in other developed countries.^4^ Unintended teen pregnancy is a public health issue with a spectrum of impacts. Teen pregnancy and birth create health,^5^ educational,^6,7^ financial,^8^ psychological,^9^ and cultural barriers to success for teen mothers and children. Teen mothers experience disparities across a wide range of health and well-being indicators.

Many of these disparities may be due to lack of alignment between postnatal behavioral health care and social services for teenage mothers that leads to poor engagement in services and subsequent poor outcomes. More than half of teen mothers experience postpartum depression, by far the highest rate of any new mothers.^10^ Disparities caused by age, ethnicity, poverty, and discrimination create barriers to medical care and receipt of social services, education, and employment.

There are significant health disparities in unintended and teen pregnancy, with poorer women and women of color experiencing significantly higher rates.^11,12^ Teens from single-parent households, those who are born to a mother who had her first child in her teen years, and those whose mothers do not have a General Education Diploma are more likely to initiate sexual activity.^4^ Hispanic males are less likely to report using contraception at first time having sex than white counterparts and Hispanic females are much more likely to rely on condom use alone (vs. hormonal methods) during their last sexual encounter.^4^ Pregnancy rates for Hispanic teenagers are at least twice as high as they are for non- Hispanic white teenagers.^9^

New Mexico (NM) has one of the highest rates of teen pregnancy in the nation, with 8% of teen women becoming pregnant in a given year.^2^ Although rates of teen pregnancy are decreasing nationally, disparate reductions have been found in NM. In NM, 84% of teen births are to Hispanic and Native American women.^13^ In our own pilot study with predominately Hispanic teens receiving primary care services in NM, 69.7% are sexually active and 36.8% report past year unprotected sex.^14^ The study explored decisions of contraception to gain cultural knowledge of external influences that effect teen pregnancy.

## Contraceptives

Long-acting reversible contraceptives (LARCs), such as implants and intrauterine devices (IUDs), are the most effective forms of birth control. LARC methods are safe, easy to use, long lasting, easily reversible, highly acceptable among patients, have limited contraindications for use, and are >20 times as effective as oral contraceptive pills.^15^ With pregnancy rates of <0.01%, LARCs have the potential to transform reproductive health care and dramatically decrease rates of unintended pregnancy. Both the American Academy of Pediatrics and the American College of Obstetrics and Gynecology recommend LARCs as the first-line contraceptive choice for adolescents who choose to be sexually active.^16^

Despite these strong recommendations, to date, only 5.8% of sexually active female teenagers nationally have used a LARC method.^4^ Among multilevel factors currently known to impact LARC use, the primary factors appear to be knowledge, access, and cost. The Contraceptive CHOICE Project demonstrated that providing women with education and access to LARCs promotes LARC use and decreases rates of unintended teen pregnancy and abortion.^16^ In this prospective cohort study, 10,000 women who wanted to avoid pregnancy for at least 1 year were read a brief script regarding LARCs and offered an array of contraception options at no cost.

Sixty-seven percent (67%) of women who received the CHOICE intervention chose an LARC method. Several factors have been found to impact uptake of LARCs across age groups, including access, cost, and knowledge provided in a health clinic setting.^17^ However, there is a gap in the current literature about LARCs and decision making among Hispanic teens and young women.

Unfortunately, little is known about the psychosocial factors that may impact decision making and serve as elements that could be targeted through intervention, especially for adolescents. A continuous premise has been that multilevel contextual factors such as cultural values, attitudes, and acculturation may impact LARC decision making, but there has been little empirical work to inform this assumption.^18^ Several qualitative studies and a few quantitative studies suggest high levels of LARC misinformation and concerns about side effects among Hispanic women.^19^

In addition, many Hispanic teens report a perceived disconnect with health systems and providers impacting their willingness to access and engage in care. Previous research suggests that Hispanic teens feel judged for their reproductive health choices and feel the single birth control option is being pushed on them.^20^

In our prior study, we demonstrated that a motivational interviewing brief intervention called TEMPO (Teens Exploring and Managing Prevention Options) delivered in a health care setting decreases rates of unprotected sex among teens at risk for unintended pregnancy.^21^ The TEMPO intervention invites teens to think critically about the impact an unintended pregnancy could have on their lives, including how it would affect important life domains such as family and social relationships, education, employment, and independence.

Such an intervention could be enhanced by increased knowledge regarding the multilevel contextual factors impacting decision making around LARCs. Knowledge about such factors could also inform the development of interventions that occur outside of health care settings across multidimensional levels of influence within the community. Rates of unintended teen pregnancy in NM, combined with disparities between Hispanic and white populations,^21^ serve as a call to action to investigate barriers and facilitators to reproductive health faced by Hispanic adolescents. Such information could be invaluable in helping generate a multilevel culturally appropriate intervention to increase LARC uptake and reduce the occurrence of unintended teen pregnancies among Hispanic teens.

## Materials and Methods

We used the theory of planned behavior (TBP) to explore perspectives and experience of Hispanic teens selecting birth control at a school-based health center. The TBP has been used previously with Hispanic teens to explore how motivations impact behavioral intentions and the proceeding behaviors.^22^ This study utilized a descriptive qualitative design.

### Recruitment

Female Hispanic teens attending a school-based clinic for first-time contraception counseling were invited to participate in a face-to-face interview after their visit, to discuss their motives for either accepting or refusing an LARC. The study recruited young women who accepted LARC and those who chose another form of contraception other than LARC. LARC can be defined as IUD or/and birth control implant.

Inclusion criteria were self-reported Hispanic ethnicity, ages 14–19 years, presenting to a primary care clinic for a first-time reproductive health care contraception counseling visit, and being able to read and speak English or Spanish. Exclusion criteria were self-reported pregnancy or pregnancy discovered during optional contraception consultation, expressed suicidality, obvious cognitive impairment, or inability to provide informed consent.

A research assistant in the waiting or examination room approached all female patients attending the clinic for reproductive health services. The research assistant asked the patient whether she was interested in learning whether she was eligible to participate in a study. Interested patients were given a brief self-administered screening instrument on an iPad using secure data capture software that assessed inclusion/exclusion criteria. Patients who were not eligible were told so and thanked for their willingness to be screened.

Participants who met eligibility criteria were informed that they may be eligible for participation and asked whether they wanted to learn about the study. After the patient's medical visit, a research assistant entered the examination room and described the study to the patient using a brief recruitment script. Patients interested in participating completed an informed consent document.

All medical contraception consultation services provided to research participants in this study were confidential and did not require parental permission consistent with the state of NM law that allows minors 13 years of age and older to receive services without consent.^2^ Participants consented to study participation on their own and a waiver of parent permission was granted by the University of New Mexico Internal Review Board (IRB). This study was submitted and IRB approved as a modification to the larger TEMPO project protocol (No. 18-375).

### Interview procedures

An interview guide was drafted and adapted with input from the study team, mentors, and a literature review focused on factors impacting LARC adoption. The study was designed and analyzed by an interdisciplinary team including teens from the community, a community member working with expectant and parenting teens, a family physician focused on adolescent health, a clinical psychologist, and public health and qualitative research experts. The guide was developed using the framework of intersectionality and integrated questions informed by socioecological concepts such as power, social policy, cultural values, income, education, occupation, family, social class, gender, and race/ethnicity.

The interview began in an open manner in which participants were asked for their main reason for choosing or not choosing an LARC. Once no new information emerged from the open questioning, we continued with the semistructured guide, to cover all aspects that might have contributed to the decision-making process.

### Analysis

Interviews were transcribed and analyzed by members of the research team. Members of the qualitative research team independently read transcripts from sets of two to three interviews, then met together to develop an initial coding structure from the transcripts. The team met on a biweekly basis to review each of the initial nominal codes collectively, identifying commonalities and resolving discrepancies resulting in a preliminary organizing scheme. Once a set of codes for the first group of transcripts was agreed on, analysts applied the organizing scheme to each of the transcripts using the conventional content analysis technique.^23^

## Results

### Description of the sample

A total of 58 adolescents agreed to be screened for the study: 20 were enrolled and 38 were ineligible. Ineligibility was due to not being Hispanic, not being within the study age range, or not attending the clinic to discuss contraception with a provider for the first time. Participants were interviewed immediately after their appointment to make birth control decisions, so their motivations and experiences are reflected in real time.

### Themes

Seven themes emerged from the analysis process. Themes were often cited as motivators for choosing a specific birth control method among a majority of participants in each group. Three themes and two subthemes were identified among both LARC and non-LARC users, two themes were found among LARC users only, and two additional themes were discussed among non-LARC users only.

## Themes Among Both LARC and Non-LARC Users

For both LARC and non-LARC users, peer influence, relationship with family, and side effects played a crucial role in birth control decisions.

### Theme 1: Peer influence

Participants almost always mentioned a friend, cousin, or sister who supported them in their contraceptive decision or educated them about different birth control options. Participants were often more likely to receive the birth control a peer had recommended or used beforehand as long as the peer's experience with the contraceptive was relatively painless and they cited it as being effective.

[my friend] told me she was on the shot. She gave me the idea to come and get the shot here at the clinic. (Participant #2) (birth control shot)

I was like nope, I'm not going to get it. But then, I talked to one of my friends and she was like no, it's a good step. She talked me through it. (Participant #21) (arm implant)

My resources are just like my experiences with the people that I've talked to. (Participant #25) (IUD)

However, misconceptions regarding the side effects of birth control were often spread through this peer influence.

[my friend] has the shot too. I talked to her about it. I was like can you let me know? She was like you are going to get hungry. You are going to start to gain weight. I was like I don't want to gain weight. (Participant #6) (birth control shot)

### Theme 2: relationships with family

Support from a peer may have been so essential to the teen's contraceptive choice because they often faced opposition from one or both of their parents. The Hispanic adolescents within our study discussed familial beliefs surrounding abstinence and premarital sex as barriers to candid conversations about sex. Most teens found it difficult to conduct these conversations with their parents or older family members because it was seldom discussed within the family and considered taboo.

I don't want to talk to them about [sex] because it's really uncomfortable for me talking to my parents. (Participant #2) (birth control shot)

Don't have sex until you are older. That's all [my parents] really tell me.. . They don't really mention birth controls or what is going to happen after. They just tell me that I'm going to have a kid and I'm not going to be able to support [it]. (Participant #8) (Plan B)

Teens who felt comfortable discussing sexual issues with their family members sometimes found support from a female relative such as an aunt, sister, or, less frequently, a mother. Feelings of wanting to live up to a parent or family member's expectations encouraged them to seek out birth control.

I really talk to my mom about everything. .. She's really the one that's impacted me. But like I said, since I am having unprotected sex, I don't want to accidentally mess up and I don't want that feeling of disappointment from my parents. (Participant #4) (arm implant)

In addition, whether or not teens were able to discuss sexual issues with their mother, they often expressed regret that their mother was unable to give them advice about a specific contraceptive option.

I think that if I was with my mom and she knew that I was getting birth control, I would have gotten that, the arm one. .. it's just safer. (Participant #6) (birth control shot)

I just really need [my mom's] approval. Like I feel that I can get any of these or whatever the doctor suggests for me. But like I need her approval. I can't do it confidential. (Participant #8) (Plan B)

### Theme 3: side effects

Both LARC and non-LARC users received misinformation about contraceptives, whether it was through a peer or the internet. Most often these misconceptions centered around side effects and the impact birth control has on the user's health.

A lot of girls complain about weight gain and their periods. You know, a lot of people are like, oh I don't have my period. That's really unhealthy. (Participant #40) (arm implant)

### Other subthemes

Hispanic teens struggle to make informed decisions about birth control due to a perceived communication disconnect between them and their clinicians, coupled with misinformation spread by peers and the internet.

I was talking about getting birth control and [my doctor] would be like well, you are at a young age. Why would you do that to your body? (Participant #45) (birth control shot)

My resources are just like my experiences with the people that I've talked to. (Participant #25) (IUD)

Because from what I heard many women that are on the pills become infertile or they have to take the pill every day and I would forget. (Participant #16) (birth control shot)

## Themes Among Only LARC Users

### Theme 4: menstrual cycle and symptoms

Those who chose an LARC discussed their menstrual cycles and period symptoms as reasons for getting the contraceptive. Often, the idea of reducing the pain of cramps or getting a lighter period was a large motivator in getting an LARC. This was more frequently mentioned among LARC users, whereas non-LARC users often mentioned their menstrual cycles in relation to confidentiality.

I care because like I don't want my mom suspecting like oh, why haven't you gotten your period? Yeah. (Participant #45) (birth control shot)

### Theme 5: clinician influence

Clinician influence was frequently mentioned in that clinicians played a pivotal role in giving reassurance to and validation of the teen's contraceptive decision. This said, a clinician could often not dissuade a teen once they chose a form of contraception.

My healthcare provider, she was really sweet and she went through everything and what everything did, how long it lasted, the positive and negatives of each and every birth control possible. (Participant #57) (arm implant)

## Themes Among Non-LARC Users

Those who chose a non-LARC option mentioned ease of protection and a need for a transition into LARC. The idea behind “transitional birth control” was that users could experiment with the hormone levels and side effects of non-LARC contraception before receiving the more daunting LARC.

### Theme 6: ease of protection

Non-LARC users discussed ease of protection in that hiding their use of birth control was cited as a crucial factor in their overall decision. Adolescents often needed to hide their use of birth control from disapproving parents who had differing beliefs about abstinence and birth control.

I wanted to get on birth control but I'm just afraid my mom is gonna find out somehow. (Participant #8) (Plan B)

The birth control I chose today was the shot.. . I spoke to my mom about having birth control.. . she said, you are not ready. .. so, I decided that maybe I should get something that won't last too long. That way she won't find out. (Participant #6) (birth control shot)

Those who mentioned ease of protection frequently discussed their concerns surrounding their periods. A birth control that made them lose their periods was not ideal because some of the participants had mothers who tracked their menstrual cycles or would notice not having to buy tampons, pads, or other sanitary supplies for their daughters.

I had a lot of concerns about my period because. .. [my mom is] usually on that like when I'm on my period. Do you need pads or do you need...? I was very concerned because my mom asks a lot about it. (Participant #6) (birth control shot)

### Theme 7: transitional birth control

The birth control shot was often cited as a useful transition into LARC use. Adolescents wanted to use birth control shot and other contraceptives to observe how their bodies would react to hormonal birth control and whether LARC would be a better option in the future.

I think it's better to get the injection first to see how I react and then I will get the implant. (Participant #16) (birth control shot)

When I run out of the pills, I might come back and get an implant or one of the IUDs. (Participant #7) (birth control pill)

## Discussion

Findings given in Table 1. demonstrate that there is no clear difference in LARC or non-LARC choice based on age. This finding was supported by qualitative data, where age did not differentiate selection of LARC versus non-LARC methods. Further research is needed to determine whether or not age has an effect on contraceptive choice. Arm implant was by far the most popular contraceptive choice, with the birth control shot second. This is most likely because both were considered to be noninvasive and easily available, whereas the IUD was often cited as a daunting choice because it requires vaginal insertion.

**Table 1.** Each Participant's Age and How Many Participants by Age Group Chose Long-Acting Reversible Contraception or Nonlong-Acting Reversible Contraception, and the Total Number of Participants in the Study

Participant age and choice of birth control						
Age	Chose LARC	% (*n* = 20)	Chose Non-LARC	% (*n* = 20)	Total	% (*n* = 20)
15	6	30%	3	15%	9	45%
16	3	15%	4	20%	7	35%
17	3	15%	1	5%	4	20%
Total	12	60%	8	40%	Total = 20 participants	

LARC, long-acting reversible contraception.

One of the major takeaways of this study is understanding motivators among Hispanic for their choice of birth control. Using the theory of planned behavior as a guide^22^ findings suggest that motivators about contraceptives include: teen attitudes, beliefs of LARC and non-LARC, family normative beliefs around abstinence and birth control, and subjective norms. School-based health clinics gave Hispanic teens autonomy and perceived control over their contraceptive selection.

Peer influence was the most frequently cited reason for contraceptive decision making among both groups. Relationships with family were barriers and supports for Hispanic teens, where some report the lack of communication and abstinence-only beliefs made it difficult to seek contraception. Hispanic teens were concerned about side effects from birth control and received misinformation from the internet and their peers, this calls for improved evidence-based culturally-centered communications about the side effects of LARC and non-LARC methods.

LARC users were concerned about their menstrual cycle and symptoms, along with clinician influences. Non-LARC users expressed transitional birth control strategies and ease or protection, where Hispanic teens could hide their use in birth control because they would have menstrual cycles.

### Limitations

Findings from this study are not generalizable to other populations or school-based health settings. A limitation is that interviews were conducted to teens who were already seeking contraceptive counseling. A public health policy hurdle is to get teens into the clinic and due to the setting of the study, the data did not capture the perspective of teens who have not scheduled an appointment. Also, a limitation is the school-based health clinic's policy of providing confidential birth control services. This may have resulted in a higher recruitment of teens who wanted to hide their birth control decisions from their parents, and their reasons for choosing LARCs may have been different than those of teens who did not use LARCs.

Social desirability bias is a limitation that we acknowledge, where Hispanic teens may have responded to interview questions based on what they viewed as most desirable by the interviewer.^24^ Another limitation is time, where recruitment and interviewing occurred during a school year and provide a description based on the students enrolled during that time period. Larger studies that explore LARC and non-LARC decision making and satisfaction with contraceptive selection are also needed. Even with these limitations, the findings fill a gap in the current contraceptive literature about Hispanic teens.

## Implications for Policy and Practice

Hispanic teen's need access to contraceptive services. Subjective and normative beliefs about sexual relationships and behaviors among Hispanic teens impact their decision-making process. Family and parent communication around sexual behavior influences teen decisions to use birth control^25^; however, in this study, parental communication was limited. In the absence of parental support, school-based confidential health centers like the one presented in this study give teens autonomy in their selection of a birth control method that is best for their bodies and lives (opposed to influences from families, friends, or providers).

Although some studies have reported discrimination within the health care setting, especially when it comes to accessing birth control, participants in this study did not report experiences of discrimination. A major strength of this study was that the interviewer was a young Hispanic woman and able to speak Spanish. Previous studies suggest that culture match is essential in the interview process with Hispanic populations.^26^ Possible future study may include developing strategies to communicate accurate and reliable information to Hispanic teens. Policies that support school-based health centers and confidential birth control must be expanded to all states, and for all populations, especially groups such as Hispanic teens who experience higher rates of unintended pregnancies.

## Conclusion

This is the first study that we are aware of that explores contraceptive selection of LARC and non-LARC methods in a Hispanic teen population in NM. Findings underscore the need for continued efforts to promote evidence-based health communication strategies and culturally matched interventions for Hispanic teens.

## Abbreviations Used

AAP: American Academy of Pediatrics
IUDs: intrauterine devices
LARC: long-acting reversible contraception
NM: New Mexico
TEMPO: Teens Exploring and Managing Prevention Options
